# Morphostructural Characterization of Rice Grain (*Oryza sativa* L.) Variety Morelos A-98 during Filling Stages

**DOI:** 10.1100/2012/940293

**Published:** 2012-05-03

**Authors:** Rosa Elena Espinosa-Mendoza, Javier Solorza-Feria, Martha Lucía Arenas-Ocampo, Brenda Hildeliza Camacho-Díaz, Alma Angélica Del Villar-Martínez, Pablo Emilio Vanegas-Espinoza, Antonio Ruperto Jiménez-Aparicio

**Affiliations:** Centro de Desarrollo de Productos Bióticos, IPN, Carretera Yautepec-Jojutla Km 6, Calle CEPROBI No. 8, San Isidro, 62731 Yautepec, Mor, Mexico

## Abstract

The morphostructure of grain rice Morelos A-98 was characterized in five stages of physiological maturation, in order to generate morphometric information during the filling process. Micrographic images from optical and scanning electron microscopy coupled to a digital capture system were used. Images were digitally processed to measure different descriptors such as shape, fractal dimension, and surface texture. Results showed that, two weeks after anthesis, an accelerated grain filling was observed, particularly on those grains positioned in the distal panicle zone, compared to those located in the base of this one. As deposition of assimilates in the grain increased, the area and perimeter of the transversal cut of the grains also increased (*P* ≤ 0.05); meanwhile, the rounded shape factor tended to increase as well (*P* ≤ 0.05), while the elliptic shape factor decreased. As the dehydrated endosperm passed from “milky” to “doughy” stages, values of fractal dimension area and endosperm perimeter as well as surface texture values showed that grain borders tended to become smoother and that there was a greater structured endosperm area (*P* ≤ 0.05).

## 1. Introduction

 Rice (*Oryza sativa* L.) is the fourth food product of basic consumption in Mexico. Mexican varieties of rice, in particular the “Morelos” one, are recognized by their agronomic, milling, and cooking characteristics [1]. Among diverse factors that determine rice commercial quality, there is uniformity in terms of size and form, cleanliness and its crystallinity or opacity. The rice variety Morelos A-98 (MorA-98) is characterized by a thin (>2.4 mm) and opaque grain of biger size (>7 mm) [2]. Opacity of MorA-98 is due to the presence of a distinctive starchy white spot in the ventral region in more than 20% of the grain surface, well known as “white belly” (WB) [2, 3]. Some authors have suggested that WB could be a defect of the grain, known as chalkiness; this discredits its general appearance and diminishes its mill yield [4–6]. 

Grains presenting chalkiness have a tendency to be less hard and more susceptible to fracture [7]. On the other hand, WB presence seems to be influenced by environmental factors, such as high temperatures, that interrupt the normal filling of the grain during its development, and therefore no uniform maturation stages occur [8]; this can also be influenced by diseases or when grain is harvested with high moisture content [5, 9]. Some authors suggest that WB is a distinguishing varietal characteristic that is controlled genetically [7]. Using differential scanning calorimetric techniques, viscoamylography and microscopy coupled to digital image analysis (DIA), it has been possible to characterize different rice varieties and the presence of WB [10, 11]. Yoshioka et al. [6] using DIA managed to establish differences between chalkiness and WB, being able to locate accurately the position of WB in the grain. Nevertheless, the structural complexity and the irregular morphology present in rice grain limit the use of analytical tools derived from Euclidean geometry for its quantification and interpretation; however, fractal geometry can allow the description of objects that exhibit a high complexity degree [12]. The fractal dimension (FD), magnitude of irregular objects, is related to diverse properties including rugosity and sinuosity [13]. Generally, FD can be expressed in linear terms as fractal dimension of perimeter (FDP) or area (FDA). FDP measures the irregularity degree of contours or borders, whereas FDA quantifies the proportion of the plane that is occupied by a given object [12]. In the case of rice, research papers are scarce on image fractal analysis; furthermore, Mexican varieties have not been object of this type of studies. In this work, optical microscopy, scanning electronic microscopy, digital image analysis, and fractal dimension were used for the morphostructural characterization of rice MorA-98 endosperm, in five stages of physiological maturation and four panicle positions; also, grain morphometric characteristics were described in relation to temperature and environmental humidity, in the probable formation of the WB in the grain of this rice variety. 

## 2. Materials and Methods

### 2.1. Biological Materials and Sampling

 The present research was carried out in an experimental parcel seeded with rice (*Oryza sativa* L.) variety MorA-98, corresponding to 2007 cycle, in the Experimental Field Zacatepec (CEZ) belonging to the National Institute of Forest, Agricultural Investigations and Livestock of Mexico (INIFAP). Plants were marked at anthesis time, obtaining grain samples on 7, 14, 21, 28, and 35 days after anthesis (DAF), at four different levels or intervals (arbitrary) in panicle from apex up to the base from the first spike. It was considered that the first level corresponded to the apex and the fourth level to the base. From each plant, three panicles were collected and 180 grains were analyzed. Panicles were kept in hermetic bags to avoid moisture losses during their transfer to laboratory. Subsequently, grains were transversely sectioned from their middle part, using a scalpel; the cut was always performed in the same direction (from the distal to the ventral part). A portion of the segmented grain was observed with an optical microscope (OM), whereas the other portion was treated with a fixation and postfixation process, in order to be observed with scanning electronic microscope (SEM), in agreement with the description made in the following sections. Finally, morphological and structural characteristics of grains were evaluated using DIA and FD, as described in further sections.

### 2.2. Environmental Data

 In order to relate temperature and environmental humidity to the formation of WB in rice variety MorA-98 throughout grain maturation in field, registries were continuously obtained recording relative humidity and maximum, minimum, and average temperatures from September 17 to October 21 of 2007; these data were provided by the meteorology station of CEZ-INIFAP.

### 2.3. Observations in Optical Microscopy and Digital Images Capture

Each cut was placed in the observation sector of the stereoscopic microscope (Nikon, model LZM 1500, Japan), making sure that the grain ventral part was orientated towards the image right side. Observations were accomplished at 3X or 4X, in such a way that all the grain transversal section was included in the observation field; illumination was realized in coaxial form, using an optical fiber system with an optical xenon illuminant (Nikon, model LI-500, Japan). Images capture was performed through a digital camera (Dage-MTI model DC330, Japan) placed in the microscope body, which was also connected by means of an interphase-type flashbus (Integral Technologies, model MV-Pro, USA) to a generic computer (processor speed 2.66 GHz and 4 GB RAM memory). Images were captured with MetaMorph V. 6.1 software (Meta Imaging Series Environment, Universal Imaging Co., USA), and they were digitally stored (1280 × 960 pixels, 300 ppp) without compression, with “tiff” format (Tagged Image File Format) until their processing with DIA. 

### 2.4. Digital Images Analysis (DIA)

WB images were digitally processed using the procedure reported by Yoshioka et al. [6]. Initially, images were standardized with the aid of Corel program PhotoPaint V11.5 (Corel Co., USA). They were converted to gray scale of 8 bits and format *.bmp (Bits map protocol), adjusting brightness, contrast, and intensity. Ensuing area of interest was manually selected, segmenting and extracting it from the rest of the image, in order to place it in a new folder, in such a way that an isolated object remained. These new images were binarized (2 bits) with the tool “Threshold”, and they were saved in *.bmp format with 300 ppp resolution for further measurement of morphometric characteristics of interest, as well as for FD evaluation.

### 2.5. Morphometric Descriptors Determination

**Figure 1:**
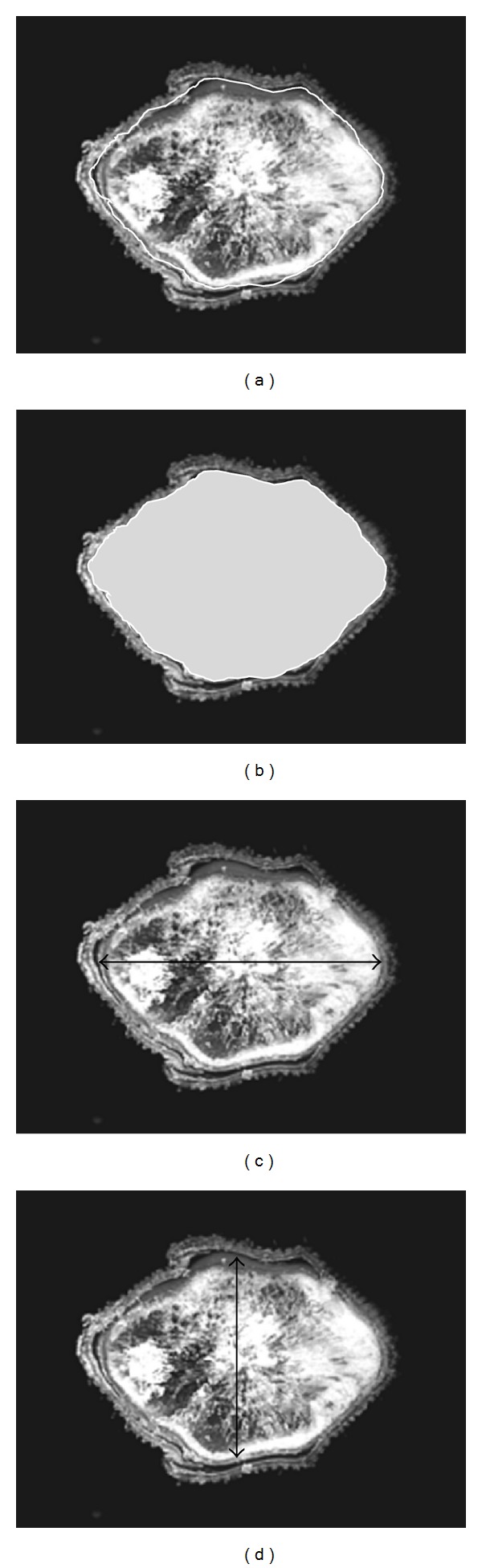
Micrographs of OM of the grain cross-section of rice Morelos A-98 (3X) showing evaluated morphometric descriptors: (a) perimeter, (b) area, (c) maximum length, and (d) minimum length.

Morphometry of images obtained by OM was evaluated with Sigma Scan Pro V. 5.0 program (SPSS Inc., USA), taking as a base the anticipated physical parameters established by the Mexican Official Norms NOM-080-SCFI-1994 and NMX-FF-035-SCFI-2005. The software was calibrated using a micrometer image (Nikon, Japan) with 100 *μ*m length, captured under the same conditions as images of rice grains. Following morphometric descriptors (Figure 1), area (A), perimeter (P), maximum length (ML), minimum length (mL), form factor (SF), and compactness factor (CF) were determined. Collected data were recorded in Microsoft Excel spreadsheet for subsequent analysis. Elliptical factor (EF) was calculated in Excel sheet, in agreement with reports by Davies [14]. ML refers to the length between two most distant pixels of object, whereas it is perpendicular to mL [15]; as a consequence, EF is the ratio of ML and mL:

(1) $$\text{EF}=\frac{\text{ML}}{\text{mL}}.$$

If such a ratio is 1.0, the object is a circle, whereas if it is >1.0, the object is an ellipse. On the other hand, SF is defined as

(2) $$\begin{array}{c} \text{SF}=\frac{4\pi \text{A}}{{\text{P}}^{2}}. \end{array}$$

Afterwards, if SF ≈ 1, the object is circular (spherical) and is not any more when SF < 1. The case of CF is a measurement of the form of an object and pertains to how compact it is or it is not; it is defined as

(3) $$\begin{array}{c} \text{CF}=\frac{{\text{P}}^{2}}{\text{A}}. \end{array}$$

If CF value is 12.57, that is to say 4*π*, the object is a compact circle; if this value is greater than this value, the object begins to take the form of a line and therefore CF tends to be infinite [15].

### 2.6. Determination of Fractal Dimension of Perimeter and Area

Fractal dimension of rice transversal cuts during its filling was calculated by means of a dimensional analysis of Power Law [16]. FDP relates characteristic length (ML) to P and measures the extension degree in which the perimeter fills the plane [13] in agreement with the equation

(4) $$\begin{array}{c} \text{P}\;\alpha \;\text{M}{\text{L}}^{\text{FDP}}, \end{array}$$

whereas FDA is determined from the relation between ML and A with

(5) $$\begin{array}{c} \text{A}\;\alpha \;\text{M}{\text{L}}^{\text{FDA}}. \end{array}$$

Clearing proportionality signs in (4) and (5), corresponding to fractal dimension values, can be obtained from respective logarithmic relations and in such a way that FDP was calculated using the equation proposed by Olsen et al. [17]:

(6) $$\begin{array}{c} \text{FDP}=2\cdot \left(\frac{\text{lnP}/4}{\text{lnA}}\right), \end{array}$$

in which FDP value quantifies the irregularity degree of the object edge, in such a way that FDP of a straight line *≈*1; if such line becomes sinuous or rough (irregular), the value will be FDP > 1.

On the other hand, for fractal area dimension FDA, the maximum area-length relationship was used, as proposed by Voss [18], according to (7).

(7) $$\begin{array}{c} \text{FDA}=\left(\frac{\text{lnA}}{\text{lnML}}\right), \end{array}$$

in which FDA value quantifies the proportion of a plane that is occupied by the object of interest in such a way that if FDA ≈ 2, the object is a plane; smaller values mean it is an incomplete plane and therefore irregular [19].

### 2.7. Scanning Electronic Microscopy (SEM)

A standard protocol was followed for fixation and postfixation of samples [20] to be observed with SEM. Transversal sections of grains (two per each sampling day, corresponding to levels 1 and 3) were introduced in vials that contained 1.5 mL of 3% (v/v) glutaraldehyde for their fixation during 2 h; three more washings with phosphate solution were undertaken, letting stand 10 min each sample, followed by dehydration with ascending ethanol concentrations (10 to 100% v/v), and then, samples were dried with 98.5% (v/v) hexamethyldisilazane. Later, treated samples were ionized with a 14 nm gold cover with 15 mAmps current. Observations were performed in a scanning electron microscope (Jeol, model JSM-5800LV, Japan).

**Figure 2:**
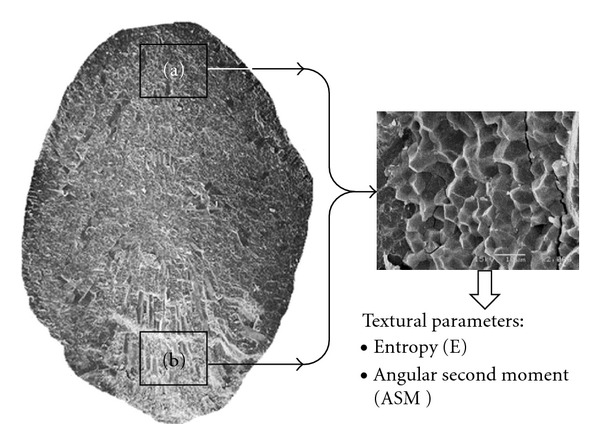
Micrograph SEM of grain transversal section of rice Morelos A-98 (500 X) showing regions (a) dorsal and (b) ventral, from which endosperm superficial texture parameters were obtained.

First a digital image was captured (640 × 480 pixels) at a 40X or 50X resolution (as required) and 20KVa of complete transversal cut, as well as two of x2,000 and 20KVa corresponding to crystalline region and WB, respectively (Figure 2).

Digital images were stored in a *.jpg format (Joint Photographic Experts Group), without using image compression. Images were converted to gray scale (8 bits, *.bmp format) in order to evaluate different textural parameters by means of DIA.

### 2.8. Evaluation of Textural Parameters

Scanning electronic micrographs (2000X magnification) were analyzed with the tool “GLMC Texture” (gray-level cooccurrence matrix) of Image J V.1.34, and, in agreement with Lepistö et al. [21], the following parameters were determined:

- Entropy (E): it corresponds to disorganization or randomness of the objects in the image that is possible to be measured based on occurrence probability of pixels, in such a way that, if these are randomly distributed (major disorganization state), E value is high; on the contrary, if pixels of objects contained in the image have a certain level of organization, the E value diminishes.
- Angular Second Moment (ASM) it corresponds to a homogeneity measurement of pixels in an image; a high value of ASM indicates that pixels that conform the objects of the image are very similar, which denotes an elevated organization level.

### 2.9. Statistical Analysis

In each of the five reproductive development stages, three panicles were randomly sampled from three independent plants. A descriptive analysis was accomplished by means of Sigma Stat V. 3.5 program (SPSS Inc., USA), which included average, median, standard deviation, standard error, maximums, and minimums. One-way analyses of variance were performed, and, in the case of obtaining significance (*P* ≤ 0.05), multiple comparative tests were realized (per week and by interval), using Dunn's test at the same significance level for evaluated descriptors in endosperm, because this test allows to make comparisons even though the same number of data is not available, as for the case of vain grains.

## 3. Results and Discussion

### 3.1. Dimensional Morphometric Descriptors

**Figure 3:**
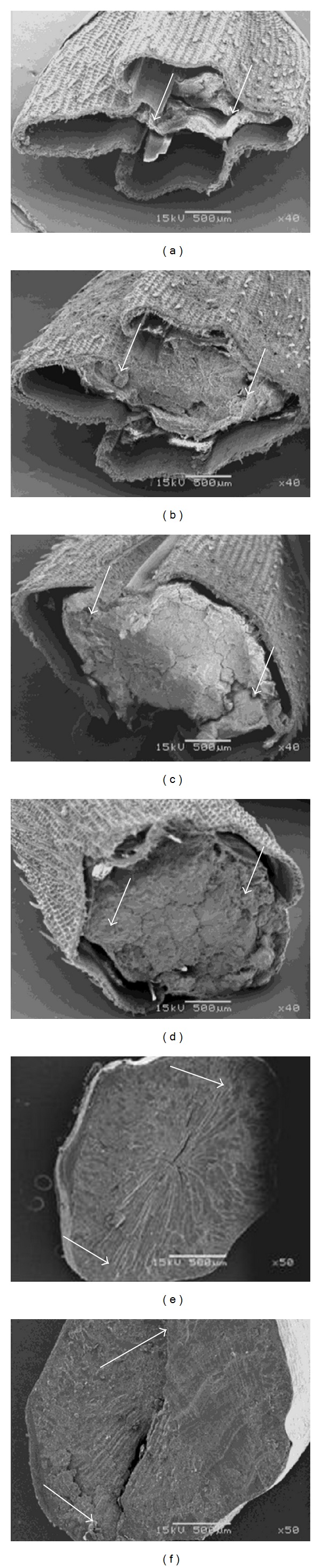
Gallery of SEM photomicrographies (transversal section) showing rice grain development of Morelos A-98: (a) 7 DAF; (b) 14 DAF; (c) 21 DAF; (d) 28 DAF; (e) 35 DAF; (f) polished grain rice. (Images(a)–(d) have a 40X magnification, whereas (e)–(f) have 50X; arrows indicate sampling position according to that established in Section 2).

**Figure 4:**
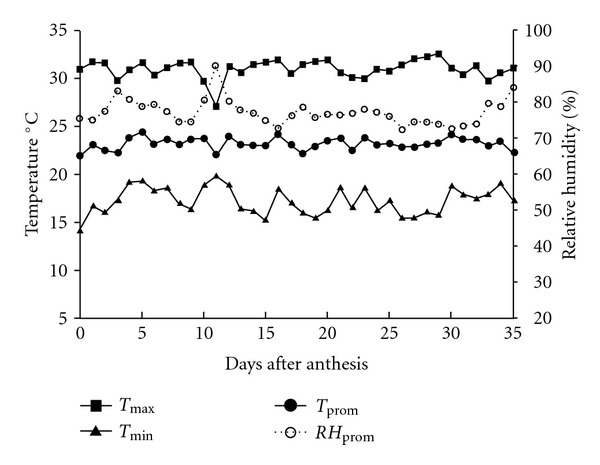
Registered temperature and relative humidity during grain filling of rice Morelos A-98 (registered data from September 17 to October 21, 2007).

With micrographs of transversal cross-section of rice grain (Figure 3), the different filling stages are exemplified, until the grain reaches its average final moisture (8–10% content). It was found that the perimeter (P), as well as the area (A) of rice grain variety Morelos A-98 increased as grain maturation advanced (Table 1). This characteristic is probably due to protein, lipid, and granule starch deposition in the embryonic grain sack [22]. Interaction between these compounds in a liquid matrix (“milky” stage) is the responsible of grain morpho-structural conformation, when this one is dried [23]. The dimensions for A and P descriptors in grains transversal cuts were statistically larger (*P* ≤ 0.05) in the higher part of the panicle, decreasing towards the base (Table 1). This suggests an asynchronous filling pattern in the first stages of grain maturation; that means materials accumulation of the endosperm took place from the apex (position 1) to the base (position 4). Previous behavior can be due, on the one hand, to the pattern in which the pollination occurs (apex to base) and, on the other hand, to the pattern in which starch synthesis occurs [24]. In this sense Mohapatra et al. [25] reported that the enzymes responsible for this process, such as the ADP-glucose pyrophosphorylase (AGP), starch synthase (SS), and their diverse isoforms, appear in an earlier way and in greater amount in grains located in the apical region, than in those located in the basal part of the panicle, which agrees with the observed changes for A and P. Besides, interval comparisons did not show significant differences between contiguous intervals for both descriptors, and, in fact, differences found tended to disappear as grain filling elapsed, for example, A in intervals 2-3 of DAF 7 or P in intervals 3-4 of DAF 21. In this form, at the end of panicle filling it has reached a degree of relatively homogenous structural development. Previous discussion agrees with data reported by Cheng et al. [26], who when characterizing diverse rice varieties found that starch in mature grains, before being harvested, had a similar structural organization. These authors postulated that the variations found depended on the panicle grain position and were related to the enzymatic activity of SS and AGP. On the other hand, comparisons realized per week for the same interval (Figure 1) showed a tendency to increase A and P during grain filling. Nevertheless, in contrast of what happened between panicle intervals, a non-uniform pattern in grain structural development was observed. That is to say, the magnitude of change in both parameters (relative speed of grain growth) was greater in the first 10 days after flowering, between DAF 7 and 14, than that obtained between the days 28 and 35, in which there was no significant difference (*P* ≥ 0.05). This pattern, apparently disordered, agrees with the finish of protein and lipid accelerated accumulation in early stages of the filling, as well as with the ending of cellular division and the endosperm expansion, to give place to the beginning of starch synthesis and deposition [22, 23]. In the same way, Mohapatra et al. [25] reported an accelerated increase in enzymes SS and AGP activity from anthesis time, reaching its maximum in 10 and 13 days after anthesis (DAF), respectively. This is relevant to the fact that, from day 21 DAF, a slight decrease in average P and A values in all the intervals was observed. This particularity coincided with the cut of irrigation water provision (day 20 DAF) and, therefore, with the decrease of grain moisture content in the accelerated endosperm solidification [8, 27]. In this process, the endosperm components shift from being a fluid (“milky” stage) to a semisolid paste (“smooth mass” stage), mainly because there is an increase in concentration of assimilates, associated with the movement and loss of water by evaporation, in the normal grain maturation process [5]. During that period, sudden changes in the environmental conditions were not observed (Figure 4). In agreement with Umemoto et al. [27] and Cheng et al. [8], drastic changes in temperature, as in environmental relative humidity, affect the normal grain filling during their development, generating nonuniform maturation stages. Between DAF 14 and 28 relative humidity oscillated between 76.3 and 78.2% (average 76.2 ± 1.3%), maximum temperature between 30.5 and 32.1°C (average 31.2 ± 0.7), and minimum temperature between 15.5 and 17.5°C (average 16.3 ± 0.7°C); that is to say, they remained without significant changes.

### 3.2. Form Descriptors

 During grain filling, the grain acquires a less elliptical form (EF value decreases) and a more rounded one (SF value increases) in its center (cross-section) (Figure 1). Although no significant differences (*P* ≥ 0.05) were found between contiguous intervals in all 35 days for both factors (except days 28 and 35 DAF), at the panicle higher part, the grain form came close to that of a circle (SF ≈ 1 and lower EF), whereas, at the panicle lower part, EF increased and FF reduced. Finally, such differences did not tend to appear as filling was increased in such a way that, between days 28 and 35, there were no significant differences for both factors (*P* ≥ 0.05). It is known that accumulation of assimilates in the rice grain appears uniformly distributed all over the grain the first days after anthesis [8, 22], filling in this way the embryonic sack, which has an elliptical form (high EF), but without exerting pressure over its walls; later, the starch accumulation occurs from the center towards the edges, forming concentric layers [22], increasing the pressure towards the embryonic sack walls, and, in addition, the moisture content decreases [5, 8]; which, overall, would give as a consequence that a rounder form would take place (SF increase).

### 3.3. Irregularity Descriptors

**Figure 5:**
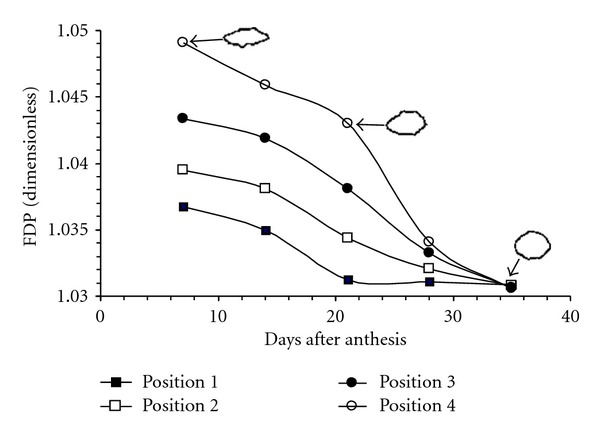
Evolution of fractal dimension of perimeter (FDP) during grain filling and maturation of rice Morelos A-98. Objects represent contour (perimeter) binarized of OM (3X) micrograph of grain transversal cut.

During rice grain filling, it was observed that FDP value decreased, making the edge of this grain less rough (Figure 5). This characteristic also appeared in relation to the panicle position, and it was placed in the lower part of the grain; that is to say, FDP of interval 2 < FDP of interval 1, and so forth. Hence, as the rice grain begins to structure, the embryonic sack walls begin to contain a greater amount of assimilates [5, 23], which would increase the turgidity pressure and, therefore, the sack edge would be smooth. The smoothness increase in the edge becomes stabilized from day 21, agreeing with the beginning of grain dehydration and solidification, until reaching the final structure in which it is harvested [8, 27]; therefore, there was a greater homogeneity in FDP values by interval in days 28 to 35.

**Figure 6:**
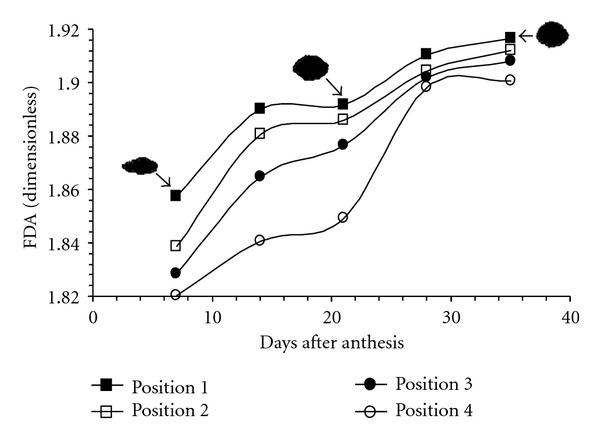
Evolution of fractal dimension of area (FDA) during grain filling and maturation of rice Morelos A-98. Objects represent binarized projected area of OM (3X) micrograph of grain transversal cut.

It was found that during the filling process of the grain as well as with the increase in its panicle position, the transversal area tended to occupy more of the plane and therefore it was less irregular (Figure 6); for instance, FDA of day 7 < FDA of day 14, and so forth and FDA of interval 2 < FDA of interval 1, and so forth. In the same way as what occurs with the embryonic sack walls, as the rice grain begins to structure itself, it displays a greater amount of assimilates [5, 22]. As a consequence of filling, the volume occupied was greater, which was observed as an increase in an occupied area in a two-dimensional image. It is important to indicate that the obtained kinetics for FDP as well as for FDA followed a nonlinear complex behavior, finding a dispersion of Power Law type, whose exponents (FDP and FDA) are fractionate, characteristic of substances migration. In this case, assimilates deposition and starch biosynthesis, may probably occur during the structuring process [28]. Results of fractal dimension were similar to those reported for other biological systems, such as plant morphology, growth of mycelium in fungi [29], growth of vegetal cells, aggregation processes of red blood cell [30], and angiostatic activity of phytomedicaments [31], whose FDP values were between 1.02 and 1.06, whereas the FDA values were from 1.7 to 1.9. 

### 3.4. Textural Parameters of MorA-98 during Endosperm Development

**Figure 7:**
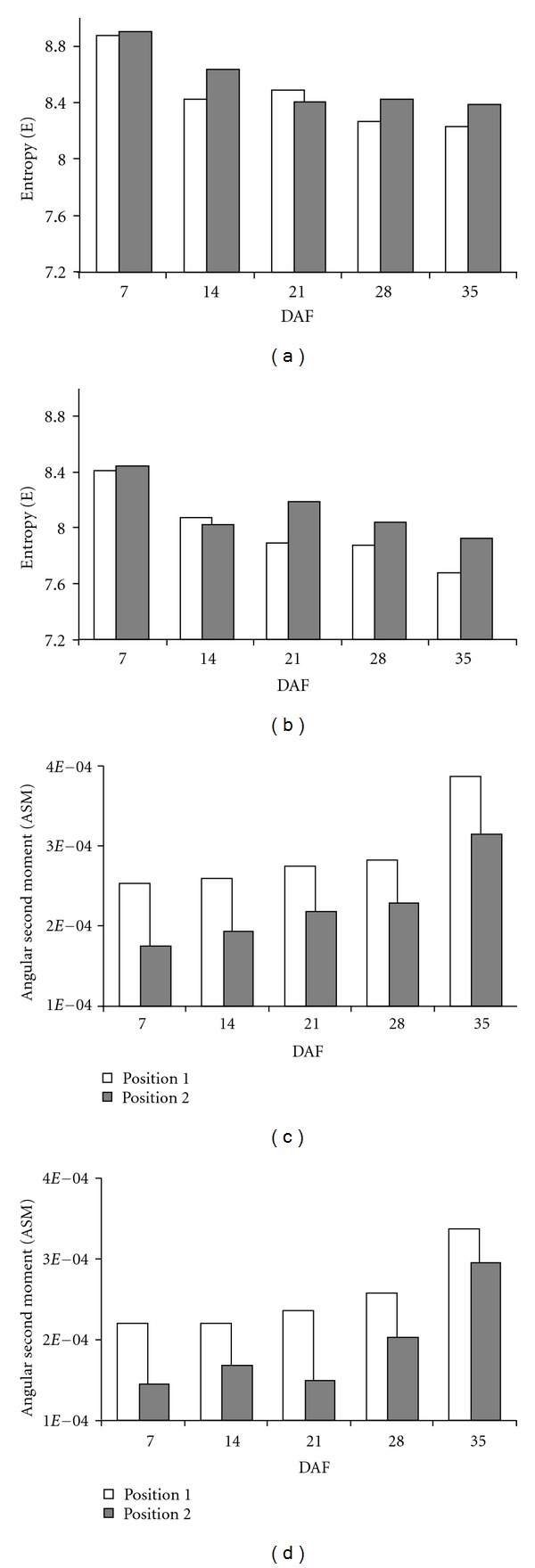
Textural parameters of electronic micrographs (2000X), corresponding to structure development of grain endosperm of rice Morelos A-98: (a,c) dorsal region and (b,d) ventral region.

Textural parameters of photomicrographs SEM, for transversal cuts of MorA-98, exhibited changes throughout the endosperm structuring, for both dorsal and ventral regions as shown in Figure 7. It was observed that the greater entropic value and, therefore, the one with highest structural irregularity appeared at the beginning of the grain filling (“milky” stage) in the dorsal region (Figure 7(a)); however, despite this situation, there were no significant differences between the different panicle positions (*P* ≥ 0.05). On the contrary, the ASM presented the smallest value at the beginning of the filling, registering significant differences between different panicle positions, in which position 1 tended to reach an ordinate structure quicker than position three. The structural level of organization/disorganization seems to become stabilized from day 21 onwards, reaching the greater value of ASM when the grain was completely dry. This behavior agrees with that reported by Cheng et al. [26], who characterized diverse varieties of rice by means of SEM, differential scanning calorimetry, and viscoamylography, finding that starch in mature grains had a similar structural organization before being harvested and that variations found depended on the grain position in panicle. With respect to textural parameters of ventral region (Figure 7(b)), the behavior was similar to that shown on dorsal region, although E and ASM values were smaller; this aspect indicates that, notwithstanding, a tendency to a structural organization of the WB exists, this one reaching a lower value than the crystalline endosperm. In the same way, these differences could be related to hardness values of the endosperm, in such a way that at a lower level of structural organization, the endosperm tends to be less hard and, thus, more susceptible to become broken [7]. It is worth mentioning that, mechanical properties (e.g., hardness), textural characteristics as well as structural organization of rice endosperm, are conferred to a great extent, by different factors, such as the starch granules morphology for the different regions (dorsal and ventral), the packing density and the occluded air spaces that are formed during grain drying [10, 32]. It would be interesting to evaluate these aspects using DIA techniques and dimension fractal for variety Morelos A-98. 

## 4. Conclusions

From the evaluation of morphometric descriptors, it was shown that filling of rice grain variety MorA-98, rapidly appears during the first 20 days after anthesis, with an asynchronous pattern of development in panicle, being higher in the apex region. In the same way, it was observed that through filling, the endosperm has a tendency to present a greater level of structural organization.

## Figures and Tables

**Table 1:** Comparison of dimensional morphometric and form descriptors by interval in transversal cut sections of rice grain Morelos A-98.

DAF	interval	A (mm^2^)	P (mm)	SF	EF	ML (mm)	mL (mm)	CF
7	1	2.36 ± 0.41*a*	8.95 ± 0.60*a*	0.52 ± 0.05*a*	2.91 ± 0.52*a*	3.09 ± 0.15*a*	1.09 ± 0.17*a*	24.65 ± 2.74*a*
	2	2.12 ± 0.52*a*	8.81 ± 0.98*a*	0.48 ± 0.07*b*	3.02 ± 0.53*a*	3.03 ± 0.32*a*	1.03 ± 0.20*ab*	26.59 ± 4.63*a*
	3	1.71 ± 0.58*b*	7.94 ± 1.33*b*	0.47 ± 0.06*b*	2.95 ± 0.51*a*	2.69 ± 0.52*b*	0.93 ± 0.20*bc*	27.03 ± 3.18*a*
	4	1.40 ± 0.50*b*	7.07 ± 1.37*c*	0.48 ± 0.05*b*	2.76 ± 0.45*a*	2.37 ± 0.50*c*	0.87 ± 0.16*c*	26.20 ± 2.81*a*

14	1	3.73 ± 0.38*a*	10.01 ± 0.55*a*	0.67 ± 0.02*a*	1.80 ± 0.15*a*	3.11 ± 0.19*a*	1.74 ± 0.14*a*	18.75 ± 0.67*a*
	2	3.56 ± 0.41*a*	9.93 ± 0.53*a*	0.66 ± 0.04*a*	1.87 ± 0.14*a*	3.16 ± 0.15*a*	1.70 ± 0.14*a*	19.23 ± 1.22*a*
	3	3.24 ± 0.52*a*	9.48 ± 0.50*b*	0.65 ± 0.06*a*	2.48 ± 0.65*b*	3.13 ± 0.13*a*	1.54 ± 0.20*b*	19.63 ± 2.00*a*
	4	1.99 ± 0.81*c*	7.87 ± 1.46*c*	0.57 ± 0.93*b*	2.26 ± 0.40*b*	2.56 ± 0.55*b*	1.15 ± 0.29*c*	22.73 ± 5.39*b*

21	1	3.65 ± 0.41*a*	9.66 ± 0.59*a*	0.71 ± 0.05*a*	1.78 ± 0.22*a*	3.02 ± 0.23*a*	1.72 ± 0.16*a*	17.80 ± 1.37*a*
	2	3.41 ± 0.55*a*	9.62 ± 0.74*a*	0.67 ± 0.07*b*	2.00 ± 0.37*ab*	3.09 ± 0.27*a*	1.59 ± 0.24*ab*	18.92 ± 1.94*a*
	3	3.06 ± 0.99*b*	9.33 ± 1.27*b*	0.61 ± 0.13*c*	2.16 ± 0.62*b*	3.04 ± 0.44*a*	1.47 ± 0.39*b*	21.61 ± 5.71*b*
	4	2.32 ± 0.91*c*	8.46 ± 1.24*c*	0.57 ± 0.11*d*	2.49 ± 0.92*c*	2.77 ± 0.40*b*	1.21 ± 0.36*c*	23.34 ± 7.37*c*

28	1	4.56 ± 0.35*a*	10.37 ± 0.46*a*	0.79 ± 0.05*a*	1.37 ± 0.07*a*	2.95 ± 0.11*a*	2.15 ± 0.10*a*	16.00 ± 1.57*a*
	2	4.50 ± 0.31*a*	10.19 ± 0.32*ab*	0.81 ± 0.03*a*	1.41 ± 0.13*a*	2.96 ± 0.11*a*	2.10 ± 0.13*a*	15.62 ± 0.55*a*
	3	4.32 ± 0.77*a*	9.99 ± 0.92*b*	0.78 ± 0.04*a*	1.46 ± 0.17*a*	2.94 ± 0.25*a*	2.05 ± 0.28*ab*	16.09 ± 0.93*a*
	4	3.61 ± 1.18*b*	9.41 ± 1.26*c*	0.71 ± 0.11*b*	1.61 ± 0.27*b*	2.83 ± 0.42*a*	1.81 ± 0.41*b*	18.49 ± 5.84*b*

35	1	4.60 ± 0.36*a*	10.27 ± 0.39*a*	0.81 ± 0.03*a*	1.38 ± 0.09*a*	2.97 ± 0.12*a*	2.16 ± 0.11*a*	15.63 ± 0.69*a*
	2	4.75 ± 0.22*a*	10.36 ± 0.29*a*	0.82 ± 0.02*a*	1.37 ± 0.06*a*	2.99 ± 0.09*a*	2.19 ± 0.07*a*	15.40 ± 0.33*a*
	3	3.96 ± 1.20*b*	9.66 ± 1.15*b*	0.75 ± 0.12*b*	1.55 ± 0.58*a*	2.87 ± 0.22*a*	1.92 ± 0.49*b*	17.28 ± 4.12*b*
	4	3.31 ± 1.35*c*	8.94 ± 1.63*c*	0.72 ± 0.12*b*	1.78 ± 0.63*b*	2.72 ± 0.40*b*	1.69 ± 0.55*b*	18.20 ± 4.00*b*

Means with same letters are not statistically different (Dunn's 0, 05); se = standard error; area (A), perimeter (P), maximum length (ML), minimum length (mL), form factor (SF) and compactness factor (CF).
